# The Malay version of SF-36 health survey instrument: testing data quality, scaling assumptions, reliability and validity in post-coronary artery bypass grafting (CABG) surgery patients at the National Heart Institute (Institut Jantung Negara—IJN), Kuala Lumpur

**DOI:** 10.1186/s12955-020-01658-9

**Published:** 2021-02-09

**Authors:** Ahmad Farouk Musa, Mohamed Shajahan Mohamed Yasin, Julian Smith, Mohd Azhari Yakub, Rusli Bin Nordin

**Affiliations:** 1 Jeffrey Cheah School of Medicine and Health Sciences, Monash University Malaysia, Bandar Sunway, Malaysia; 2 Department of Surgery, School of Clinical Sciences at Monash Health, Monash University, Clayton, Australia; 3 Department of Cardiothoracic Surgery, Monash Health, Melbourne, Australia; 4 Department of Cardiothoracic Surgery, National Heart Institute, Kuala Lumpur, Malaysia; 5 School of Medicine, Faculty of Health and Medical Sciences, Taylor’s University, Subang Jaya, Malaysia

**Keywords:** Validity, Reliability, Internal consistency, Cronbach alpha, Composite reliability, Confirmatory factor analysis (CFA), Convergent validity, Discriminant validity, Malay version of SF-36, Data quality, Scaling assumptions

## Abstract

**Background:**

The Short Form 36 (SF-36) is a scoring system comprising of 36 items categorized into eight constructs corresponding to patients’ health-related quality of life. It has been used extensively in various countries on different sub-populations and used to indicate the health status and help to ascertain the effect of clinical interventions on the particular population.

**Objective:**

To examine the psychometric properties of the Malay version of SF-36 (Malay SF-36) summated rating scales and validate the scale among post-coronary artery bypass grafting surgery (CABG) patients at the National Heart Institute (IJN), Kuala Lumpur.

**Methods:**

Five hundred and nine post-CABG patients at the IJN, Malaysia completed the questionnaires between 1 July and 31 December 2017. Psychometric tests endorsed by the “International Quality of Life Assessment Project” were utilised.

**Results:**

The data quality was excellent with a high questionnaire completion rate (100%). As hypothesized, the ordering of item means within scales was clustered. In unison, scaling assumptions were satisfied. Good discriminant validity was shown between subsets of patients with various levels of health status. Notwithstanding, there were probably translation issues of the Physical Functioning scale which showed small ceiling effects. We clearly observed high ceiling and floor effects in both Role Physical and Role Emotional scale most probably attributed to the dichotomous style of their choice of responses. Cronbach alpha values of the eight scales ranged from 0.73 to 0.90, showing good internal consistency reliability. Confirmatory Factor Analysis (CFA) confirmed the 8-factor solution and Composite Reliability revealed internal consistency reliability except for Vitality and Social Functioning. Based on the Average Variance Extracted (AVE), convergent validity was adequate except for two domains. Discriminant Validity is good for the eight constructs as the √AVE are generally higher than the correlation coefficients between the latent constructs.

**Conclusion:**

The scoring for the Malay SF-36 based on the summated ratings method was proven to be valid to be applied in our local clinical population. The CFA, fitness estimates, reliability and validity assessments suggest that the Malay version of SF36 is a valid and reliable instrument. However, further work is warranted to further refine the convergent validity and reliability of some scales.

## Introduction

The SF-36 Health Survey was first validated in Malaysia by Sararaks et al. [1] in 2004 on asthmatic patients but its use as a quality of life (QOL) health survey instrument in this country has been generally limited. Often, it is assumed that once a questionnaire has been validated, it would also be valid and reliable amongst other group of patients. However, when a detailed assessment is conducted, it is frequently discovered not to be the case [2, 3]. The outcome scores might be different and perhaps meaningless if this process of validation is not undertaken within a specific group. Considering the fact that we plan to institute this Malay SF-36 Health Survey to our patients undergoing coronary artery bypass grafting (CABG) surgery at the National Heart Institute (IJN) [4] and would be using the Malay version of SF-36 which we have translated, it would be imperative to validate this Malay version of SF-36, and to ensure that we retained the psychometric properties for valid interpretation of the SF-36 scores in this translation process.

The SF-36 has been translated into many languages including some East Asian languages such as Chinese, Japanese, Thai and Vietnamese. Several studies [5–10] have tested the reliability and construct validity of the Thai SF-36 and concluded that it was reliable and valid for assessing QOL in Thailand. However, other Asian translation of the SF-36 reported some problems that were manifested in the psychometric tests. In the Chinese and Japanese translations, discriminant validity, especially between the concepts of mental health and vitality, was problematic [11, 12]. Similarly in the Vietnamese translation, some problems were detected with the internal consistencies in several of the scales. Cultural differences in definition or the structure of health might have been the reason for these problems.

Despite the issues regarding the translation, it has to be acknowledged that SF-36 has attractive characteristics in measuring health status. It is one of the most comprehensive health questionnaires and has been validated across the different ages and covering many major health concepts. The general nature of the questionnaire rather than being specific to any particular disease conditions made it possible to be used widely in many circumstances. Due to the increasing use of this questionnaire in this country, we feel there is a need to validate this questionnaire again.

## Aims

The main aim of this study was to examine the psychometric properties of the SF-36 summated rating scales and to report on the reliability and validity of the translated Malay version of SF-36 in a clinical population of post-CABG patients at the National Heart Institute, Kuala Lumpur, Malaysia.

## Methods

### Study design

This study took place at the Cardiothoracic Clinic, National Heart Institute of Kuala Lumpur from July until December 2017 on patients who had undergone surgery not more than 6-months period from the time of the interview. Initially the English version of SF-36 was translated into Malay by one translator and then was back-translated into English by another translator. The back-to-back translation was done and verified by two certified linguists including a native English speaker who masters both languages before we proceed with the survey.

The Malay SF-36 and the socio-demographic questions were administered to the subjects. We administered the questionnaire via interview taking into consideration that this will enhance the patients’ understanding of the questionnaires and would be able to minimize any missing responses. Responses were coded for each item which were then summed up and transformed into a scale from 0 to 100. Zero representing the worst possible health status and a hundred the best possible health status. Missing values were then substituted in accordance to the guidelines suggested by the developers [13].

### Sampling method

Sampling was purposeful as the main aim was to validate the Malay version of SF36.

## Sample size calculation

Sample size calculation was based on the estimation of the population mean, standard deviation of the outcome of interest, confidence level, and the pre-specified margin of error according to Sullivan [14]. According to the formula for sample size calculation below, the bigger the standard deviation of the outcome of interest, the bigger is the sample size obtained, assuming that the confidence level and margin of error are constant:

$${\text{n}} = [({\text{Z}}_{{{1} - \alpha /{2}}} \cdot \sigma )/{\text{E}}]^{{2}}$$

where n = sample size, Z_1−α/2_ = confidence level (usually 1.96), σ = standard deviation of the outcome of interest, E = pre-specified margin of error or precision (usually 0.05).

Based on the above consideration, therefore, the computed sample size according to the highest standard deviation (SD) of the eight dimensions of SF36—PF (Physical Functioning), RP (Role Limitation—Physical), BP (Bodily Pain), GH (General Health), VT (Vitality), SF (Social Functioning), RE (Role Limitation − Emotional), MH (Mental Health)—will yield the largest sample size.

From the above table (Table 1), the highest SD is 46.2 (RE). Since the highest expected population standard deviation is 46.2, and employing the formula above, the study would require a sample size of 328 to estimate a mean with 95% confidence and a precision of 5%. In other words, if we select a random sample of 328 from a population, and determine the mean (y) to be 60.5, we would be 95% confident that the mean in the population lies somewhere between y − 5 and y + 5 (60.5 − 5 and 60.5 + 5): 55.5 and 65.5. Calculation of the sample sizes based on other SDs of the remaining seven dimensions (lower than 46.2) yielded sample sizes lower than 328. Therefore, the minimum sample size required in this study is 328.

**Table 1:** Descriptive statistics of score distributions for SF-36 scales: combined sample [14]

	PF	RP	BP	GH	VT	SF	RE	MH
Mean	77.1	66.6	67.3	57.2	62.7	79.2	60.5	65.7
SD	25.0	42.1	31.5	20.0	26.9	46.2	24.4	24.4
Minimum	5	0	0	5	5	0	04	4
Maximum	100	100	100	100	100	100	100	100
% floor	0	22	0.5	0	0	0.5	33	0
% ceiling	28	55	39	3	8	49	65	6

*PF* physical functioning, *RP* role limitation—physical, *BP* bodily pain, *GH* general health, *VT* vitality, *SF* social functioning, *RE* role limitation—emotional, *MH* mental health

## Ethical statement

The National Heart Institute Research Ethics Committee approved the study (IJNREC 359/2017). No amendment was made throughout the duration of the study. We also registered the study with the National Medical Research Register (NMRR-17-2763-39427), Ministry of Health on 27th December 2017.

## Coding of items and scales

The Malay SF-36 is a questionnaire consisting of 36 items which were clustered into eight health concepts which are meant to be measured. The health concepts are Physical Functioning (PF), Role Limitations due to Physical Health (RP), Bodily Pain (BP), General Health Perception (GH), Vitality (VT), Social Functioning (SF), Role Limitations due to Emotional Problems (RE), and Mental Health (MH). There is also one additional single-item measure of Health Transition (HT).

### Statistical analysis

A pilot study was carried out on thirty patients to determine the face validity. We utilised SPSS version 24.0 to analyse the data set. Descriptive analysis was carried out to describe the distribution of data according to mean and standard deviation.

We expected the highest mean scale scores when measuring disability (PF, RP, BP, SF, RE) and lower mean scores when measuring the well-being range (GH, VT, MH). Scale scores should have substantial variability in order for a scale to include all important levels. For each scale, we calculated the ceiling scores (score of 100) and the floor scores (score of 0). In order for the scale to capture the full range of potential responses in the population, the ceiling and floor effect should be less than 20% each [15].

We examined the missing and out-of-range data which were normally associated with translation problem in order to evaluate the summated ratings scale. Evaluation of data completeness and response distribution was characterized by generating descriptive statistics. Cronbach’s α coefficient was a test of internal consistency reliability and construct validity was investigated to determine the extent to which scores correlated with criteria based on theory [16].

To confirm the hypothesized scale structure, we used tests of scaling assumptions to determine whether items are assembled as a same construct within a scale, indicating items of a scale can be summed without weights to generate scale scores and also examining the item-scale correlations [17]. These tests were to look into the item internal consistency and item discriminant validity. We consider item internal consistency to be substantial and satisfactory if the correlation between an item and its hypothesized scale is noted to be at least 0.40. For item discriminant validity to be successful, the correlation between an item and its own scale must be higher, at least by two standard errors, as compared to the other scales. After the scale-level analyses were examined after performing the item-level analyses as described above, we constructed the summated rating scales.

We undertake the Confirmatory Factor Analysis (CFA) using the maximum likelihood method (AMOS 24.0) to test the factorial validity of the original model [18]. The fit of the model was evaluated using a number of indices. Non-significant (*p* > 0.05) values of χ^2^ indicate acceptable fit, but this statistic is sensitive to sample size, i.e. in larger samples, the value tends to be significant. Jöreskog and Sörbom [19] suggested using χ^2^/*df* to address this problem, and Ullman [20] proposed χ^2^/*df* < 2.0 as the criterion of acceptable fit. We also calculated the following indices of fit: comparative fit index (CFI) and goodness of fit index (GFI)—values range from 0 to 1 and values larger than 0.90 indicate adequate fit [21] although more recently, Hu and Bentler [22] suggested > 0.95 as the criterion for good fit; root mean square error of approximation (RMSEA)—values lower than 0.08 indicate adequate fit [23].

In CFA, reliability was determined by computing the composite reliability (CR) statistic. In exploratory research, values of composite reliability between 0.60 and 0.70 are acceptable, while in more advanced stages, the value have to be higher than 0.70 [24]. Convergent validity was analysed by comparing the average variance extracted (AVE) for each factor with the factor’s correlation with other constructs and discriminant validity was evaluated by comparing the square root of AVE (√AVE) and the square of the correlation between the factors. To establish convergent validity, the factor loading of the indicator, CR and the AVE have to be considered [24]. The value ranges from 0 to 1. AVE value should exceed 0.50 so that it is adequate for convergent validity [25]. To assess discriminant validity, we used the Fornell–Lacker criterion [26] by comparing the square root of the AVE with the correlation of latent constructs [24]. A latent construct should explain better the variance of its own indicator rather than the variance of other latent constructs. Therefore, the square root of each construct’s AVE should have a greater value than the correlations with other latent constructs [24].

## Results

Five hundred and nine post-CABG patients were recruited into the study. The median age of the subjects was 59 years with a range from 29 to 83. The majority (84.1%) were males and were predominantly of the Malay race (70.1%). Indian race made up of 17.7%, Chinese 9.8% and the other races made up the rest. The majority were hypertensive (81.2%), almost half were diabetic (53.7%), and almost two-third (72.4%) have hypercholesterolemia. Almost half of these patients (41.1%) had a pre-operative NYHA Class I, and 53.9% of them are of Class II. Almost all patients (99%) had on-pump CABG and only 10.4% had CABG and valve surgery at the same time.

### Data quality

There were no missing items. All response choices were used.

#### Ordering of items means

We noticed that the ordering of item means (Table 2) was congruous with what we hypothesised from the health hierarchy. As observed from the PF section, the highly challenging item which is PF1 (vigorous exercise) recorded the lowest mean whereas PF10 (bathing) had the highest mean. As hypothesized, the results also showed that item means were reduced across clusters as hypothesised. Patients had significant limitations (lower mean score) in walking more than a kilometre (PF7) as compared to 100 m (PF8).

**Table 2:** Malay SF-36: item percent missing, item means and standard deviations (SD)*

Scale	SF-36 item	Code	% missing	Mean	SD
Physical Functioning (PF)	Q3. Vigorous activities	PF1	0	1.89	0.688
	Q9. Moderate activities	PF2	0	2.24	0.712
	Q6. Walking more than a kilometre	PF7	0	2.25	0.744
	Q8. Climbing several flights of stairs	PF4	0	2.29	0.651
	Q5. Bending, kneeling, stooping	PF6	0	2.35	0.723
	Q4. Walking more than 100 m	PF8	0	2.36	0.692
	Q10. Lifting or carrying groceries	PF3	0	2.37	0.683
	Q7. Walking 100 m	PF9	0	2.49	0.700
	Q11. Climbing one flight of stairs	PF5	0	2.53	0.656
	Q12. Bathing or dressing	PF10	0	2.66	0.679
Role-Physical (RP)	Q14. Accomplished less than would like	RP2	0	1.52	0.500
	Q16. Difficulty performing work/activities	RP4	0	1.52	0.500
	Q13. Cut down time spent on work	RP1	0	1.56	0.497
	Q15. Limited in kind of work/activities	RP3	0	1.59	0.492
Bodily Pain (BP)	Q21. Extent pain interfered with work	BP2	0	2.13	1.011
	Q22. Intensity of bodily pain	BP1	0	2.72	1.234
General Health (GH)	Q1. My health is excellent	GH5	0	1.99	1.001
	Q36. I seem as healthy as anyone I know	GH3	0	2.09	1.007
	Q34. Rating of general health	GH1	0	2.75	0.847
	Q33. I seem to get sick easier than others	GH2	0	3.59	1.291
	Q35. I expect my health to get worse	GH4	0	4.09	1.078
Vitality (VT)	Q27. Full of life	VT1	0	2.41	1.240
	Q23. Have a lot of energy	VT2	0	2.59	1.325
	Q29. Feel tired	VT4	0	4.00	1.432
	Q31. Feel worn out	VT3	0	4.76	1.441
Social Functioning (SF)	Q20. Extent health problems interfered	SF1	0	1.81	0.960
	Q32. Frequency health problems interfered	SF2	0	3.92	0.987
Role-Emotional (RE)	Q18. Accomplished less than would like	RE2	0	1.69	0.462
	Q17. Cut down time spent on work	RE1	0	1.73	0.444
	Q19. Work not done as carefully as usual	RE3	0	1.83	0.372
Mental Health (MH)	Q26. Felt calm and peaceful	MH3	0	2.19	1.284
	Q30. Been a happy person	MH5	0	2.19	1.314
	Q24. Been a very nervous person	MH1	0	4.55	1.571
	Q28. Felt down hearted and blue	MH4	0	4.91	1.491
	Q25. Felt down in the dumps	MH2	0	5.27	1.291
Health Transition (HT)	Q2. Change in health from one year ago	HT	0	2.09	0.941

^*^Items within a scale are ordered from lowest to highest according to their relative expected means

Looking at the VT scale, we noticed that items measuring energy and welfare (VT1 and VT2) had lower means than items measuring tiredness and exhaustion (VT3 and VT4) as we hypothesised. Similarly, when we look at the MH scale, it was clearly apparent that the items measuring positive effects (MH3 and MH5) had lower means as compared to those that measure negative effects (MH1, MH2, MH4).

We hypothesised that the two role functioning elements, which enquired patients whether they achieved less (RP2 and RE2) had the lowest mean. While we observed this for the RE2 within the RE scale, the RP2 within the RP scale did not have the lowest mean.

We observed that the mean score for Health Transition element was 2.09, which means that they have a slightly worse health status as compared to a year ago.

#### Tests of scaling assumption

We noticed for BP, GH, VT, SF and MH (scales with 5- and 6-choice response) the standard deviations were almost identical and close to 1.0. Table 3 summarizes the result for item-scale correlation.

**Table 3:** Malay SF-36: item-scale correlations

Scale	Item									
PF	PF1	PF2	PF3	PF4	PF5	PF6	PF7	PF8	PF9	PF10
	0.494**	0.702**	0.732**	0.764**	0.770**	0.735**	0.790**	0.810**	0.828**	0.540**

RP	RP1	RP2	RP3	RP4						
	0.709**	0.847**	0.848**	0.842**						

BP	BP1	BP2								
	0.941**	0.911**								

GH	GH1	GH2	GH3	GH4	GH5					
	0.501**	0.334**	0.441**	0.204**	0.488**					

VT	VT1	VT2	VT3	VT4						
	0.443**	0.450**	0.527**	0.560**						

SF	SF1	SF2								
	0.500**	0.539**								

RE	RE1	RE2	RE3							
	0.859**	0.867**	0.638**							

MH	MH1	MH2	MH3	MH4	MH5					
	0.612**	0.597**	0.323**	0.549**	0.289**					

*PF* Physical Functioning, *RP* role limitations due to Physical Health, *RE* role limitations due to Emotional Problems, *VT* Vitality, *MH* Mental Health, *SF* Social Functioning, *BP* Bodily Pain, *GH* General Health

^**^Spearman correlation is significant at the 0.01 level (2-tailed)

For all but two scales (PF and MH), correlation of elements with their respective hypothesised scales were generally similar. The item-scale correlation of all elements were 0.08 units or less from at least one other item-scale correlation within its scale, excepting the item-scale correlations of PF1 and MH5 which were 0.494 and 0.289 units respectively from the next closest item correlations in their scales. All item-scale correlations were greater than 0.40 except for: GH2 (0.334), GH4 (0.204), MH3 (0.323), and MH5 (0.289). We observed success rate of 100% for the item internal consistency test for six scales except for GH (94.30%) and MH (94.30%) (Table 4).

**Table 4:** Malay SF36: Tests of scaling assumptions

Scale	No. of items per scale (k)	Item internal consistency			Item discriminant validity		
		Range (correlation coefficients)^a^	Comparison^b^	Success rate (%)	Range (correlation coefficients)^c^	Comparison^d^	Success rate (%)
PF	10	0.49–0.83	10/10	100	0.00–0.40	70/70	100
RP	4	0.71–0.85	4/4	100	0.01–0.53	28/28	100
BP	2	0.91–0.94	2/2	100	0.02–0.46	14/14	100
GH	5	0.20–0.50	3/5	60	0.02–0.44	33/35	94.30
VT	4	0.44–0.56	4/4	100	0.02–0.42	28/28	100
SF	2	0.50–0.54	2/2	100	0.06–0.60	14/14	100
RE	3	0.64–0.87	3/3	100	0.01–0.55	21/21	100
MH	5	0.29–0.61	3/5	60	0.01–0.34	33/35	94.30

*PF* Physical Functioning, *RP* role limitations due to Physical Health, *RE* role limitations due to Emotional Problems, *VT* Vitality, *MH* Mental Health, *SF* Social Functioning, *BP* Bodily Pain, *GH* General Health

^a^Correlations between items and hypothesized scale, corrected for overlap

^b^Number of items out of k with correlation ≥ 0.40

^c^Correlations between items and other scales

^d^Number of items out of 7 × k where difference between the correlation of the item with its own scale and correlation with the other scales ≥ 2SE (= 0.0576)

#### Scale properties

We observed that as hypothesised, scales that gauged both positive and negative aspects of welfare (GH, VT and MH) scored lower means than those that are measuring disablement (PF, RP, BP, SF and RE) (Table 5). There was also a wide spread of distribution of scores where a complete full range (0–100) was shown in six out of the eight scales (Table 4). We foresaw the distributions to be towards the course of positive health observed by the high median and negative skewness since these are post-CABG patients. The comparatively low mean of 67.20 for PF was also expected since these are middle-aged patients. And this was also shown in the low ceiling effect of 9%.

**Table 5:** Malay SF36: descriptive statistics for the eight scales

Scale	Range	Median	Mean	SD	Skewness	% floor	% ceiling
PF	0–100	70.00	67.20	24.85	− 0.652	1.0	9.0
RP	0–100	50.00	54.86	40.39	− 0.166	24.0	34.6
BP	10–100	67.50	68.68	23.14	− 0.221	0.4	20.2
GH	0–100	70.00	69.27	19.31	− 0.470	0.0	4.1
VT	0–100	70.00	68.80	18.87	− 0.392	0.0	5.7
SF	0–100	75.00	76.38	20.80	− 0.683	0.0	26.1
RE	0–100	100.00	75.25	33.96	− 1.003	8.3	59.7
MH	8–100	80.00	77.39	18.68	− 0.666	0.0	15.9

Since the scores were negatively skewed, we have used the median and interquartile range (IQR) as descriptive statistics in this study

*PF* Physical Functioning, *RP* role limitations due to Physical Health, *RE* role limitations due to Emotional Problems, *VT* Vitality, *MH* Mental Health, *SF* Social Functioning, *BP* Bodily Pain, *GH* General Health

There were few patients that scored the lowest scale level, which is also known as the floor effect. We observed the floor effects in less than 1 in 100 of the respondents for all scales except for RP and RE scales. Inter alia these scales (RP and RE) also exhibited high ceiling effect (34.9% and 59.7%, respectively). And minimal floor effects and ceiling effects are also observed in scales that measure both disablement and welfare (GH, VT and MH).

#### Reliability

In five of the eight scales, the inter-scale correlation and internal consistency reliability (Cronbach alpha) coefficient estimates surpassed that of the 0.7 level as endorsed for group comparisons [27]. The reliability estimate for the other three was marginally low. For VT scale it was 0.64, SF scale was 0.63 and MH scale was 0.69, which was just below the 0.7 criterion (Table 6).

**Table 6:** Malay SF36: Inter-scale correlations and internal consistency reliability (Cronbach α coefficients, on the diagonal, in bold)

	PF	RP	BP	GH	VT	SF	RE	MH
PF	**0.90**							
RP	0.439**	**0.83**						
BP	− 0.449**	− 0.491**	**0.83**					
GH	− 0.059	− 0.041	0.134**	**0.78**				
VT	− 0.002	0.002	0.049	0.164**	**0.64**			
SF	0.000	− 0.013	− 0.001	− 0.006	0.132**	**0.63**		
RE	0.251**	0.574**	− 0.381**	− 0.040	− 0.024	− 0.061	**0.71**	
MH	0.186**	0.103*	− 0.051	0.229**	0.416**	− 0.055	0.165**	**0.69**

*PF* Physical Functioning, *RP* role limitations due to Physical Health, *RE* role limitations due to Emotional Problems, *VT* Vitality, *MH* Mental Health, *SF* Social Functioning, *BP* Bodily Pain, *GH* General Health

^**^Correlation is significant at the 0.01 level (2-tailed)

^*^Correlation is significant at the 0.05 level (2-tailed)

#### Validity

We observed that scales representing similar constructs (MH and VT) (0.416) as compared to those of competing construct (PF and RE) (0.251) have higher coefficient between scales. We also observed that most of the inter-scale correlation coefficients were generally low except for between VT and MH where it was slightly higher (0.416), and for inter-scale correlation between RP and PF (0.439), BP and PF (− 0.449), and BP and RP (− 0.491).

#### Confirmatory Factor Analysis

**Fig. 1:**
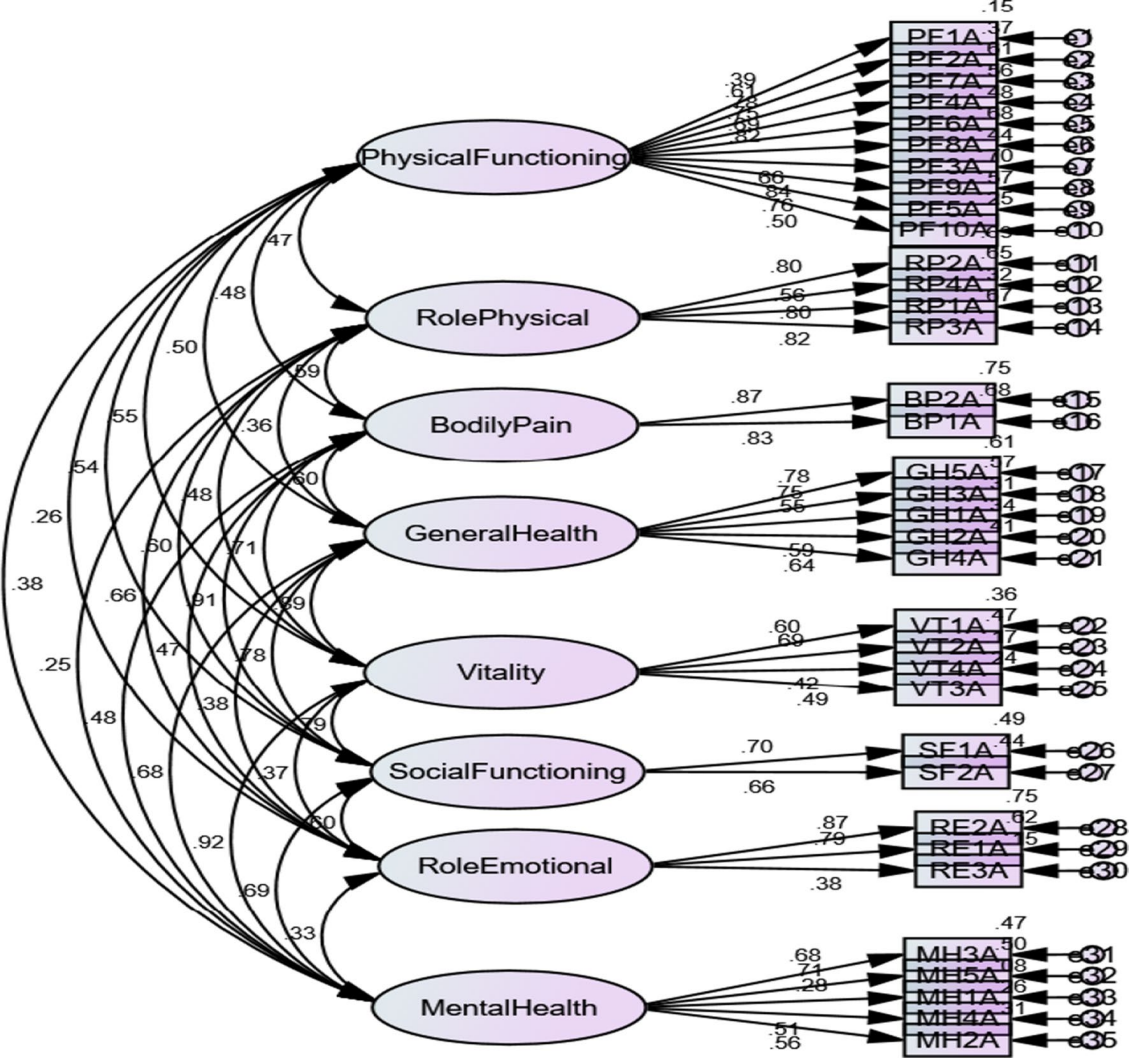
SF36 (Malay version): Confirmatory Factor Analysis and fit indices of the model to the data. Model fit indices: (χ^2^ = 2054.81, *df* = 532, χ^2^/*df* = 3.86, p < 0.000), GFI = 0.79, AGFI = 0.75, NFI = 0.77, TLI = 0.80, CFI = 0.82, and RMSEA = 0.075

Confirmatory Factor Analysis (CFA) confirmed this 8-factor solution and the fit indices generally support the fit of the model to the data: χ^2^ = 2054.81, *df* = 532, χ^2^/*df* = 3.86, p < 0.000), GFI = 0.79, AGFI = 0.75, NFI = 0.77, TLI = 0.80, CFI = 0.82, and RMSEA = 0.075 (Fig. 1).

Composite reliability (CR) revealed, except for two domains that have CR below 0.7 [Vitality (0.6) and Social Functioning (0.6)], that the remaining six domains [Physical Functioning (0.9), Role Physical (0.8), Bodily Pain (0.8), General Health (0.8), Role Emotional (0.7) and Mental Health (0.7)] have adequate convergence or internal consistency. Based on the average variance extracted (AVE), convergent validity was clearly not adequate for two of the domains [Vitality (0.3) and Mental Health (0.30]. Discriminant validity is generally adequate for the eight constructs as the values of the square root of AVE (√AVE) are generally higher than the correlation coefficients between the latent constructs (Table 7).

**Table 7:** Composite reliability (CR), the square root of the average variance extracted (AVE) (in bold) and correlations between constructs (off-diagonal)

Latent constructs	CR	AVE	Latent constructs							
			A	B	C	D	E	F	G	H
Physical Functioning: A	**0.9**	**0.5**	**0.7**							
Role Physical: B	**0.8**	**0.6**	0.465	**0.8**						
Bodily Pain: C	**0.8**	**0.7**	0.481	0.594	**0.8**					
General Health: D	**0.8**	**0.5**	0.5	0.36	0.601	**0.7**				
Vitality: E	**0.6**	**0.3**	0.552	0.485	0.707	0.889	**0.6**			
Social Functioning: F	**0.6**	**0.5**	0.543	0.596	0.911	0.776	0.787	**0.7**		
Role Emotional: G	**0.7**	**0.5**	0.264	0.663	0.472	0.382	0.373	0.602	**0.7**	
Mental Health: H	**0.7**	**0.3**	0.382	0.254	0.478	0.677	0.916	0.692	0.328	**0.6**

## Discussion

The main aim of this study was to validate the Malay SF-36. We have shown that the psychometric properties of the Malay SF-36 were satisfactory and that the summated ratings could be used for SF-36 scoring. The overall data quality was satisfactory with a very high completion rate and no missing data perhaps due to the method used in obtaining data by interview instead of self-administered survey. The questionnaire also demonstrated good face validity.

When we look at the ordering of item means, they were clustered as hypothesized except for a few in RP2 and GH3. Given the dichotomous nature of RP2, we noticed only a small deviation. The deviation of GH3 is also observed in other studies [28, 29]. The way GH3 was constructed in that it measures health in relation to other people, was attributed to explain this deviation, while absolute health was measured by the construction of GH1 and GH5.

The hypothesized scaling scale structure of the Malay SF-36 and use of summated ratings algorithm were supported by the results of scaling scale assumption. However, the item-scale correlations of RE3 to the other RE items did not fully satisfy the scaling scale assumption. And also MH3 to other MH items. These discrepancies were probably not significant problems since similar discrepancies were also seen in other studies [27, 30].

There were areas that warrant further investigation. As compared to the mean scale scores of RP and BP, the mean score of PF was higher. And the ceiling effect was noticeably lower at 9% when in other sample the ceiling effect was greater than 20% [27].

In this study, there was also a high ceiling effect in the two role functioning scale of RP (34.6%) and RE (59.7%). Perhaps the dichotomous format of the items comprising these scales could explain this finding. We have observed a similar findings in other studies as well such as when a comparison was made by Gandek and Ware [28] in 11 countries that showed a ceiling effect that ranges from 63.05 to 82.9% for RP and 69.0% to 82.8% for RE.

The internal consistency reliability was generally acceptable for group level comparison although it is relatively low in the SF scale (0.63). However, we found similar results in other studies in Asia as well. The Taiwanese translation reported reliabilities of 0.39 [31], the Chinese translation reported 0.57 [32] and 0.65 [33], the Vietnamese translation reported 0.67 [29], and the Thai translation reported 0.55 [34].

In our study, the correlations within-scale were generally higher than correlations between scales. Meaning that, there is a discrimination between the different concepts being measured. And we did not notice any higher correlations between VT and MH items compared to other Asian studies [30, 34, 35]. This result was attributed to the cultural norms of many Asians in that happiness and a healthy mental state is considered central to the concept of vitality. The same phenomenon is also seen in the Thai translation [34] and the authors attributed this to the fact that as a Buddhist country, a healthy mental state is principally fundamental to vitality. Considering this issue, Chang et al. [36] suggested to organize the items along the dimensions of well-being and distress in order for the vitality (VT) and mental health (MH) items to be more meaningful.

### Limitations

Despite being the pioneer to validate the Malay SF-36 among the post-CABG patients, this study is limited to adults who had undergone CABG and able to comprehend the Malay language, which is the National language of this country, and widely spoken by all the different races. However there is still a possibility of response bias though we have been able to minimize the possibility of response bias by conducting an interview method survey rather than a self-administered questionnaire.

### Future directions

Though the usage of SF-36 in this country is still limited, it is being increasingly accepted as a standard QOL measure in health surveys in the United States and many parts of the world. Further work should examine the properties of the scale in a larger population sample in this country, among those suffering from Ischaemic heart disease, and also among the general population at large.

## Conclusion

Our study has provided evidence that the summated ratings method could be used for scoring the Malay SF-36 and supports the use of this instrument in our population. Although reliability and validity were established for use of the instrument, there is a need for future endeavour to further improve the reliability and discriminant validity of some of the scales.

## Data Availability

All deidentified Raw data and the Output Data are provided including the supplementary files consisting of the Malay SF-36, Participant Information Sheet both in English and Malay, and the Consent Form both in English and Malay.

## Abbreviations

BP: Bodily Pain
CABG: Coronary artery bypass grafting
GH: General Health Perception
IJN: Institut Jantung Negara
MH: Mental Health
PF: Physical Functioning
QOL: Quality of Life
RP: Role limitation due to Physical Health
RE: Role limitations due to Emotional Problems
SF: Social Functioning
SF-36: Short Form 36
VT: Vitality
